# Gut microbiota signatures of long-term and short-term plant-based dietary pattern and cardiometabolic health: a prospective cohort study

**DOI:** 10.1186/s12916-022-02402-4

**Published:** 2022-06-15

**Authors:** Zelei Miao, Wenwen Du, Congmei Xiao, Chang Su, Wanglong Gou, Luqi Shen, Jiguo Zhang, Yuanqing Fu, Zengliang Jiang, Zhihong Wang, Xiaofang Jia, Ju-Sheng Zheng, Huijun Wang

**Affiliations:** 1 College of Life Sciences, Zhejiang University, Hangzhou, China; 2 Key Laboratory of Growth Regulation and Translational Research of Zhejiang Province, School of Life Sciences, Westlake University, 18 Shilongshan Rd, Cloud Town, Hangzhou, China; 3 Chinese Center for Disease Control and Prevention, National Institute for Nutrition and Health, Beijing, China; 4 Key Laboratory of Trace Element Nutrition, National Health Commission, Beijing, China; 5 Westlake Intelligent Biomarker Discovery Lab, Westlake Laboratory of Life Sciences and Biomedicine, Hangzhou, China; 6 Institute of Basic Medical Sciences, Westlake Institute for Advanced Study, Hangzhou, China

**Keywords:** Plant-based dietary pattern, Gut microbiota, Prospective cohort, Cardiometabolic health, Food frequency questionnaire, 3-day 24-h dietary recalls

## Abstract

**Background:**

The interplay among the plant-based dietary pattern, gut microbiota, and cardiometabolic health is still unclear, and evidence from large prospective cohorts is rare. We aimed to examine the association of long-term and short-term plant-based dietary patterns with gut microbiota and to assess the prospective association of the identified microbial features with cardiometabolic biomarkers.

**Methods:**

Using a population-based prospective cohort study: the China Health and Nutrition Survey, we included 3096 participants from 15 provinces/megacities across China. We created an overall plant-based diet index (PDI), a healthful plant-based diet index (hPDI), and an unhealthful plant-based diet index (uPDI). The average PDIs were calculated using repeat food frequency questionnaires collected in 2011 and 2015 to represent a long-term dietary pattern. Short-term dietary pattern was estimated using 3-day 24-h dietary recalls collected in 2015. Fecal samples were collected in 2015 and measured using 16S rRNA sequencing. We investigated the association of long-term and short-term plant-based dietary patterns with gut microbial diversity, taxonomies, and functional pathways using linear mixed models. Furthermore, we assessed the prospective associations between the identified gut microbiome signatures and cardiometabolic biomarkers (measured in 2018) using linear regression.

**Results:**

We found a significant association of short-term hPDI with microbial alpha-diversity. Both long-term and short-term plant-based diet indices were correlated with microbial overall structure, whereas long-term estimates explained more variance. Long-term and short-term PDIs were differently associated with microbial taxonomic composition, yet only microbes related to long-term estimates showed association with future cardiometabolic biomarkers. Higher long-term PDI was associated with the lower relative abundance of *Peptostreptococcus*, while this microbe was positively correlated with the high-sensitivity C-reactive protein and inversely associated with high-density lipoprotein cholesterol.

**Conclusions:**

We found shared and distinct gut microbial signatures of long-term and short-term plant-based dietary patterns. The identified microbial genera may provide insights into the protective role of long-term plant-based dietary pattern for cardiometabolic health, and replication in large independent cohorts is needed.

**Supplementary Information:**

The online version contains supplementary material available at 10.1186/s12916-022-02402-4.

## Background

With the recent advocate of the planetary health diet, plant-based dietary pattern has attracted more and more attention from both scientific community and the public, given that it shows beneficial effects not only for human health but also for the environmental sustainability [1, 2]. Prior epidemiological evidence suggests that plant-based foods are associated with lower risk of cardiometabolic diseases, such as type 2 diabetes [3], cardiovascular disease, and obesity [4], while red meat is associated with higher risk of cardiometabolic diseases [5]. Plant protein, replacing animal protein, in the substitution model, is associated with a lower risk of cardiovascular mortality [6]. Recommendations for higher intake of plant foods, such as whole grains, fruits, vegetables, legumes, seeds, and nuts, are widely integrated into different dietary guidelines globally [7]. Gut microbiota provides a key connection between diet and metabolic health [8]. Although it is reasonable to postulate that plant-based dietary pattern may influence the gut microbiota profiles, evidence from a large longitudinal cohort is still rare. Identification of key gut microbiota signatures of the plant-based dietary pattern may help reveal novel mechanistic insight into the preventive role of plant foods for cardiometabolic diseases.

Previous literature linking diet and gut microbiota mainly relies on food frequency questionnaire (FFQ), which evaluates the long-term habitual dietary intake [8, 9]. There are few large-scale human cohorts using multiple 24-h dietary recalls investigating the association of relatively short-term diet intake with gut microbiota profiles. A combination of FFQ and 24-h dietary recalls may help comprehensively characterize long-term and short-term dietary intake, which is important for the understanding of the interplay between diet and gut microbiota. Moreover, longitudinal cohort linking plant-based (long-term or short-term) diet-related microbiota features with future cardiometabolic risk factors has been rare, although there have been some reports from cross-sectional studies [8, 10].

**Fig. 1:**
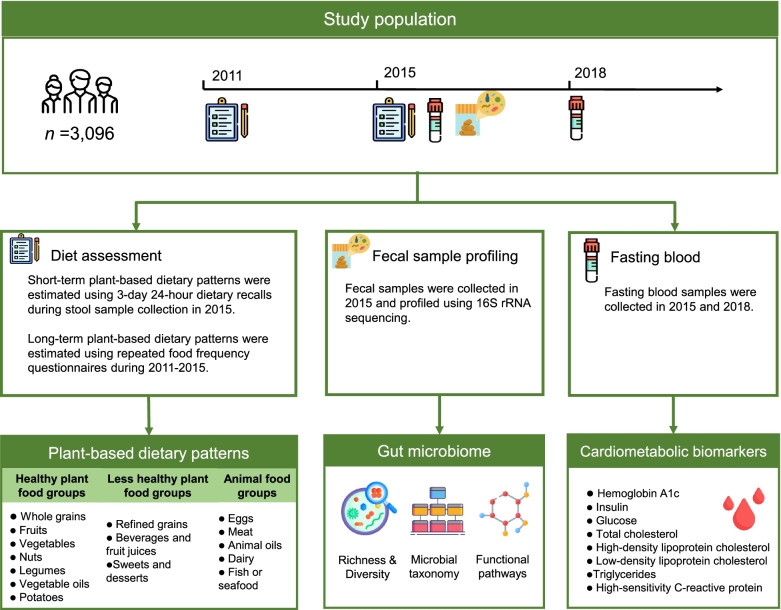
Overview of the study and analysis workflow. This study profiled the gut microbiome of 3096 participants from the China Health and Nutrition Survey (CHNS) via 16S rRNA sequencing. The CHNS has repeatedly collected dietary information using 24-h dietary recalls for three consecutive days and validated food frequency questionnaires (FFQs) in 2011 and 2015. We associated gut microbial diversity, taxonomies, and pathways with long-term plant-based dietary pattern and short-term plant-based dietary pattern, respectively. We further investigated the prospective associations between the identified gut microbiome signatures and cardiometabolic biomarkers assessed in 2018. We compared the results obtained from long-term plant-based dietary pattern and short-term plant-based dietary pattern

Therefore, in the present study, we aimed to examine the associations of long-term habitual plant-based dietary pattern repeatedly assessed over 4 years, with gut microbiota profiles in a population-based prospective cohort study in China. As a comparison, we also investigated the association of short-term plant-based dietary pattern, estimated using 3-day 24-h dietary recalls, with gut microbiota profiles in the same cohort. We further examined the longitudinal associations of the identified plant-based dietary pattern-related gut microbial features with the cardiometabolic risk factors measured after a 3-year follow-up (Fig. 1).

## Methods

### Populations and study design

Our present study was based on data from the China Health and Nutrition Survey (CHNS). The CHNS is an ongoing, prospective, household-based cohort survey of 11 rounds (1989–2018). The identical multistage, stratified, random cluster sampling scheme was used to draw samples from 12 provinces and three megacities that vary in demography, geography, economic activity, and public resources, as previously described [11]. The CHNS protocol was approved by the Institutional Review Boards of the Chinese Center for Disease Control and Prevention (No. 201524), University of North Carolina at Chapel Hill and the National Institute for Nutrition and Health (No. 07–1963). All participants signed written informed consent forms. This study followed the Strengthening the Reporting of Observational Studies in Epidemiology (STROBE) reporting guideline for cohort studies [12].

In the present study, 3249 stool samples collected in 2015 were selected for 16S rRNA sequencing. To examine the relationship between the long-term plant-based dietary pattern and gut microbiome, we included 3096 adult participants in the statistical analysis, after excluding participants without FFQ information during 2011 and 2015 (*n* = 15), participants who had gastrointestinal diseases (Crohn disease, ulcerative colitis, irritable bowel syndrome, *n* = 26), or those who had used antibiotics less than 3 months before the stool collection (*n* = 112). To link short-term plant-based dietary pattern with gut microbiome, we included 3066 participants who completed 3-day 24-h dietary recalls during stool sample collection in the current analysis.

### Dietary assessment and covariate collection

Dietary data were collected using both FFQs and three consecutive 24-h dietary recalls in each survey year. In 2011 and 2015, we repeatedly used FFQ to collect participants’ habitual diet information during the past 12 months. The FFQ consisted of 74 food items in 2011 and 63 food items in 2015. For each food item, participants were asked to estimate their food consumption frequency and amount with the help of a trained interviewer. The participants also completed 24-h dietary recalls on three consecutive days, including two weekdays and one weekend day. Additionally, the individual consumption of oil and condiment was calculated using a household weighing method.

We collected detailed demographic, medical, and lifestyle data via standard questionnaires. Total physical activity was estimated from self-reported 7-day recalls of occupational, transportation, domestic, and leisure activities. The urbanization index was calculated based on community-level physical, social, cultural, and economic environments [13].

### Plant-based diet indices

We calculated an overall plant-based diet index (PDI), a healthful plant-based diet index (hPDI), and an unhealthful plant-based diet index (uPDI) based on 15 food groups [14]. Healthy plant food groups included whole grains, fruits, vegetables, nuts, legumes, vegetable oils, and potatoes, while less healthy plant food groups included refined grains, beverages and fruit juices, and sweets and desserts. Animal food groups included eggs, meat, animal oils, dairy, and fish or seafood. The 15 food groups were created and classified based on the Chinese Food Composition Table 2002 [15] and the Chinese Dietary Guideline 2016. The details of food items constituting the 15 food groups were shown in Additional file 1 Table S1. We classified potatoes into healthy plant foods, as many Chinese would consume potatoes as a replacement of refined grains in a non-fried manner (steam or stew with meat and other vegetables). Compared with isocaloric refined grains, non-fried potato consumption was associated with better diet quality [16]. Carbohydrate intake from vegetables (including potatoes) is recommended over that from refined grains according to the Chinese Dietary Guideline 2016. We did not include tea or coffee for the indices’ creation, as these data were not available for the 3-day 24-h recalls. We, therefore, adjusted for the habitual tea and coffee consumption in the statistical models.

All food groups were divided into quintiles. The range of each food group by quintiles was provided in Additional file 1 Table S2. For the PDI, plant food groups were assigned by ascending values (1 to 5) based on the intake quintiles, whereas animal food groups were assigned by descending values (5 to 1). PDI was the sum of these values, ranging from 15 (lowest possible score) to 75 (highest possible score), and a higher score represented more plant foods consumed. For the hPDI, ascending values were given to healthy plant food groups and descending values to less healthy plant food groups and animal food groups. For the uPDI, ascending values were applied to less healthy plant food groups and descending values to healthy plant food groups and animal food groups. We calculated average values of the PDIs using FFQ data collected in 2011 and 2015 to reflect the long-term plant-based dietary pattern. Short-term plant-based dietary pattern was estimated using 3-day 24-h dietary recalls during fecal sample collection in 2015. We considered all three indices (PDI, hPDI, uPDI, in quintiles) as the main exposure variables.

### Gut microbiota profiling

Details on stool sample collection, microbial DNA extraction, and paired-end 16S rRNA gene sequencing were described previously [17]. Briefly, in the 2015 study visit, stool samples from adult participants aged 18–80 years were collected. All the participants received detailed illustrated instructions describing how to collect and store the stool samples. The V4 region of 16S rRNA gene was sequenced using the Illumina HiSeq PE-250 platform (Illumina Inc., USA). Taxonomic and functional profiles were generated using QIIME2 (version 2019.10) [18]. Pair-end reads were assembled using qiime tools import command. Low-quality regions of the sequences, marker gene Illumina sequences, and chimeric sequences (“consensus”) were filtered using the DADA2 pipeline. Reads were then summarized to amplicon sequence variants (ASV) in a feature table and annotated using the Naïve Bayes classifier trained on the Sliva_132 99% OTUs reference databases. Four alpha-diversity indices were calculated at the sampling depth of 6000: Shannon’s diversity index, observed features, Pielou’s measure of species evenness, and Faith’s phylogenetic diversity. We performed functional prediction from the ASV table using the PICRUSt2 algorithm [19].

### Measurement of cardiometabolic risk factors

Fasting blood samples after an overnight fast of at least 8 h were collected in both study visits of 2015 and 2018. Serum glucose was measured using the glucose oxidase phenol 4-aminoantipyrine peroxidase kit (Randox Laboratories Ltd, UK). Hemoglobin A1c (HbA1c) was measured by high-performance liquid chromatography system (HLC-723 G7, Tosoh Co., Japan). Fasting insulin was determined by radioimmunology in a gamma counter using an XH-6020 analyzer (North Institute of Bio-Tech, China). Serum high-density lipoprotein cholesterol (HDL-C), low-density lipoprotein cholesterol (LDL-C), total cholesterol (TC), and triglycerides (TG) were measured using the glycerol-phosphate oxidase method and polyethylene glycol (PEG)- modified enzyme assay (Kyowa Medex Co., Ltd, Japan) on the automatic analyzer (Hitachi 7600, Hitachi Inc., Japan). High-sensitivity C-reactive protein (CRP) was measured by the immunoturbidimetric method with commercial reagents (Denka Seiken, Japan) on an automatic analyzer (Hitachi 7600, Hitachi Inc., Japan).

### Statistical methods

Difference in cardiometabolic health biomarkers was tested across different quintiles of PDIs using ANOVA. We estimated the correlations between the long-term and short-term PDIs using the Spearman correlation. As a primary analysis, we examined the associations of the long-term and short-term PDIs (by quintiles) with gut microbial diversity, taxonomies and pathways using linear mixed regression. All four alpha-diversity indices (Shannon’s diversity index, observed features, Pielou’s measure of species evenness, and Faith’s phylogenetic diversity) were standardized to *Z*-score values before statistical analysis. In the linear mixed regression model, we included the following covariates: age, sex, body mass index (BMI), total energy intake, physical activity, education level, current smoking status, current alcohol drinking status, habitual tea and coffee consumption, urbanization index, sequencing depth, and sequencing batch. We further included stool sampling location in the linear mixed model to adjust the heterogeneity of the gut microbiota composition among the provinces or megacities. Given the potential correlations of the sequencing data within different sequencing batches and sampling locations [20, 21], we included sequencing batch and sampling location as random effects in the linear mixed models. A random intercepts model was used with unstructured variance–covariance matrix and maximum likelihood methods. Given that prevalent disease and related medication use were identified as important covariates of microbiome-related association studies [22], we also conducted a sensitivity analysis with additional adjustment for prevalent hypertension, hypertension medicine use, prevalent type 2 diabetes, and related medicine use. We calculated the Bray–Curtis dissimilarity metrics for each sample using taxonomic data at ASV level. We then performed permutational multivariate analysis of variance (PERMANOVA) to assess the associations between the PDIs and overall microbial structure and quantify the percentage of variance in microbial composition explained by each PDI. The above multivariable model was adjusted for age, sex, BMI, total energy intake, physical activity, education level, current smoking status, current alcohol drinking status, habitual tea and coffee consumption, urbanization index, sequencing depth, sequencing batch, and sampling location.

For taxonomic and functional features, we first filtered out all ASVs, genera, phylum, and pathways with a mean relative abundance of < 0.01% and a prevalence of < 10%. In order to account for the non-normal distribution of the microbiome data, we transformed relative abundances of the microbial features that met the inclusion criteria using rank-based inverse normal transformation before further analysis. At the phylum level, we focused on *Bacteroidetes*, *Firmicutes*, and *Bacteroidetes* to *Firmicutes* ratio, as available evidence suggested that these two phyla and their ratio were substantially affected by plant-based diet [23]. We used linear mixed models to examine the associations between PDIs and microbial taxonomies and functional pathways, adjusted for age, sex, BMI, total energy intake, physical activity, education level, current smoking status, current alcohol drinking status, habitual tea and coffee consumption, urbanization index (fixed effects), and sampling location (random effect). Multiple comparisons were controlled by false discovery rate (FDR, *q* < 0.25). We also explored another FDR threshold of 0.15 to test the robustness of our findings. Subsequently, we analyzed the associations with gut microbial diversity, taxonomies, and pathways for each food group intake with a linear mixed model adjusted for the same covariates as the above PDIs.

We then quantified the prospective associations of the identified microbial signatures with cardiometabolic risk biomarkers after a 3-year follow-up (in 2018) using linear regression models. We included eight cardiometabolic risk biomarkers (fasting glucose, HbA1c, insulin, HDL-C, LDL-C, TC, TG, and CRP). The above linear regression models were adjusted for age, sex, BMI, and corresponding cardiometabolic risk biomarkers measured in 2015. Significant association for each cardiometabolic biomarker was expressed as the difference in standard deviation (SD) and reported at *q* < 0.25 level. The statistical analyses were performed using Stata 15 (StataCorp, Texas, USA) or R (version 3.6.3).

## Results

### Population characteristics

In 2015, the mean (± SD) PDI were 45 ± 5 and 46 ± 6 for long-term diet and short-term diet, respectively. Participants with a higher long-term or short-term PDI score were less likely to be smokers or alcohol drinkers (Table 1). The food components of the plant diet index were correlated with each other at weak to moderate magnitudes (Spearman correlation coefficient ranging from − 0.33 to 0.50).

**Table 1:** Characteristics of participants during stool sample collection by quintiles of plant-based diet index^a^

	Long-term PDI (N = 3096)			Short-term PDI (N = 3066)		
	Q1 (N = 737)	Q3 (N = 506)	Q5 (N = 596)	Q1 (N = 638)	Q3 (N = 665)	Q5 (N = 520)
Age (year)	52.0 (11.4)	52.1 (13.1)	50.5 (13.1)	50.3 (11.6)	51.0 (12.2)	53.9 (13.1)
Sex, % of women	329 (45%)	267 (53%)	353 (59%)	231 (36%)	357 (54%)	334 (64%)
BMI (kg/m^2^)	23.9 (3.5)	24.2 (3.5)	24.5 (3.7)	23.9 (3.4)	23.8 (3.5)	24.6 (3.7)
Education level						
Middle school or lower	506 (69%)	328 (65%)	398 (67%)	384 (60%)	423 (64%)	383 (74%)
High school or professional college	151 (20%)	112 (22%)	122 (20%)	172 (27%)	150 (23%)	89 (17%)
University	80 (11%)	66 (13%)	76 (13%)	82 (13%)	92 (14%)	48 ( 9%)
Current smoking	256 (35%)	116 (23%)	149 (25%)	245 (38%)	175 (26%)	101 (19%)
Current alcohol drinking	251 (34%)	128 (25%)	161 (27%)	249 (39%)	182 (27%)	111 (21%)
Physical activity (MET•hours/week)	152.3 (155.0)	136.3 (135.0)	156.7 (158.3)	148.7 (145.6)	149.0 (159.2)	141.1 (142.6)
Total energy intake (kcal/day)	2186 (1190)	2183 (3475)	2174 (2441)	2585 (611)	1906 (533)	1404 (454)
Urbanization^b^						
Low	243 (33%)	156 (31%)	238 (40%)	170 (27%)	217 (33%)	245 (47%)
Middle	256 (35%)	183 (36%)	186 (31%)	241 (38%)	216 (32%)	140 (27%)
High	238 (32%)	167 (33%)	172 (29%)	227 (36%)	232 (35%)	135 (26%)
Prevalent hypertension	86 (12%)	77 (15%)	93 (16%)	74 (12%)	82 (12%)	97 (19%)
Hypertension medicine use	69 (9%)	65 (13%)	76 (13%)	61 (10%)	69 (10%)	77 (15%)
Prevalent type 2 diabetes	78 (11%)	67 (13%)	67 (11%)	73 (11%)	73 (11%)	66 (13%)
Type 2 diabetes medicine use	14 (2%)	18 (4%)	14 (2%)	13 (2%)	16 (2%)	15 (3%)
Fasting glucose (mmol/L)	5.6 (1.6)	5.6 (1.8)	5.4 (1.3)	5.6 (1.8)	5.5 (1.4)	5.4 (1.4)
HbA1c (%)	5.7 (1.0)	5.8 (1.1)	5.7 (0.9)	5.8 (1.1)	5.7 (0.8)	5.8 (1.0)
Fasting insulin (μU/mL)	7.8 (7.8)	7.7 (6.9)	7.5 (6.5)	7.9 (8.8)	7.8 (7.9)	7.8 (6.9)
HDL-C (mmol/L)	1.3 (0.3)	1.3 (0.4)	1.3 (0.3)	1.3 (0.3)	1.3 (0.3)	1.3 (0.3)
LDL-C (mmol/L)	3.3 (0.9)	3.2 (0.9)	3.0 (0.9)	3.3 (1.0)	3.2 (0.9)	3.1 (0.9)
TC (mmol/L)	5.1 (1.0)	5.0 (1.1)	4.7 (1.0)	5.1 (1.1)	4.9 (1.0)	4.8 (1.0)
TG (mmol/L)	1.6 (1.2)	1.6 (1.2)	1.5 (1.0)	1.7 (1.2)	1.6 (1.0)	1.5 (1.1)
CRP (mg/L)	1.8 (3.5)	2.1 (3.7)	1.6 (3.0)	1.9 (3.6)	1.7 (2.7)	2.1 (4.2)
Current tea drinking^c^	343 (47%)	239 (47%)	238 (40%)	331 (52%)	308 (46%)	182 (35%)
Current coffee drinking^c^	55 (7%)	20 (4%)	65 (11%)	66 (10%)	59 ( 9%)	23 ( 4%)
hPDI	40.4 (5.0)	45.1 (5.0)	50.4 (4.7)	41.3 (4.1)	45.2 (4.2)	48.6 (3.7)
uPDI	47.1 (7.3)	44.7 (7.7)	43.3 (6.9)	46.0 (6.4)	44.6 (5.4)	44.8 (3.9)
Whole grains, servings/day	0.2 (0.7)	0.3 (1.0)	0.4 (0.6)	0.3 (0.9)	0.5 (0.9)	0.6 (1.1)
Fruits, servings/day	0.6 (0.6)	1.1 (1.3)	1.4 (1.2)	0.3 (0.6)	0.4 (0.7)	0.5 (0.7)
Vegetables, servings/day	2.6 (2.0)	3.0 (2.2)	3.5 (4.0)	2.7 (1.3)	2.8 (1.4)	3.3 (1.6)
Nuts, servings/day	0.5 (1.1)	0.9 (1.4)	1.4 (1.9)	3.1 (10.3)	3.7 (11.0)	3.4 (10.4)
Legumes, servings/day	0.9 (1.8)	1.1 (0.9)	1.8 (7.4)	0.6 (0.9)	0.6 (0.9)	0.9 (1.0)
Potatoes, servings/day	0.2 (0.3)	0.4 (0.6)	0.7 (0.6)	0.2 (0.4)	0.3 (0.4)	0.5 (0.6)
Vegetable oils, servings/day	2.2 (2.1)	3.0 (2.9)	3.1 (1.9)	1.4 (2.5)	1.8 (2.2)	2.3 (2.5)
Refined grains, servings/day	7.0 (2.3)	6.8 (4.5)	6.3 (2.3)	6.9 (2.7)	7.2 (2.9)	8.6 (3.0)
Beverages and fruit juices, servings/day	0.4 (1.2)	0.8 (1.9)	1.5 (5.9)	0.1 (0.5)	0.2 (2.1)	0.2 (1.4)
Sweets and desserts, servings/day	0.3 (0.5)	0.5 (0.7)	0.7 (0.9)	0.4 (1.7)	0.6 (1.7)	0.5 (1.2)
Dairy, servings/day	0.4 (0.9)	0.4 (0.7)	0.3 (0.7)	0.1 (0.3)	0.1 (0.2)	0.0 (0.1)
Eggs, servings/day	0.7 (0.5)	0.7 (0.6)	0.6 (0.6)	0.7 (0.6)	0.5 (0.5)	0.4 (0.5)
Fish or seafood, servings/day	0.8 (3.3)	0.5 (0.8)	0.4 (0.6)	0.8 (1.2)	0.5 (0.8)	0.2 (0.5)
Meat, servings/day	2.5 (1.6)	1.9 (1.2)	1.3 (0.9)	2.7 (1.6)	2.3 (1.6)	1.3 (1.5)
Animal oils, servings/day	0.8 (1.4)	0.4 (0.7)	0.2 (0.4)	1.0 (2.2)	0.5 (1.2)	0.3 (0.7)

*Abbreviations*: *BMI* Body mass index, *CRP* High-sensitivity C-reactive protein, *HDL-C* High-density lipoprotein cholesterol, *hPDI* Healthy plant-based diet index, *LDL-C* Low-density lipoprotein cholesterol, *PDI* Plant-based diet index, *Q* Quintile, *TC* Total cholesterol, *TG* Total triglycerides, *SD* Standard deviation, *uPDI* unhealthy plant-based diet index

^a^Data are presented as mean (SD) for continuous measures, and *n* (%) for categorical measures. Plant-based dietary index, dietary intakes and total energy presented were calculated based on the FFQs, and 3-day 24-h dietary recalls for long-term diet and short-term diet, respectively. Dietary intake for each food group was adjusted for total energy intake using the residual method

^b^Urbanization index, a 12-component scale based on community-level physical, social, cultural, and economic environments, was categorized as low (≤ 63), middle (63–84.3), and high (> 84.3) levels of urbanization

^c^Current tea and coffee drinking status were estimated based on the FFQ information

The mean levels (± SD) of fasting blood glucose, Hb1Ac, insulin, HDL-C, LDL-C, TC, TG, and CRP were 5.54 ± 1.55 mmol/L, 5.74 ± 0.98%, 7.85 ± 7.76 μU/mL, 1.27 ± 0.33 mmol/L, 3.20 ± 0.91 mmol/L, 4.98 ± 1.01 mmol/L, 1.58 ± 1.19 mmol/L, 1.86 ± 3.65 mg/L, respectively. Participants who had a higher long-term PDI had lower LDL-C and TC. Similar trend was observed for short-term PDI (Table 1).

**Fig. 2:**
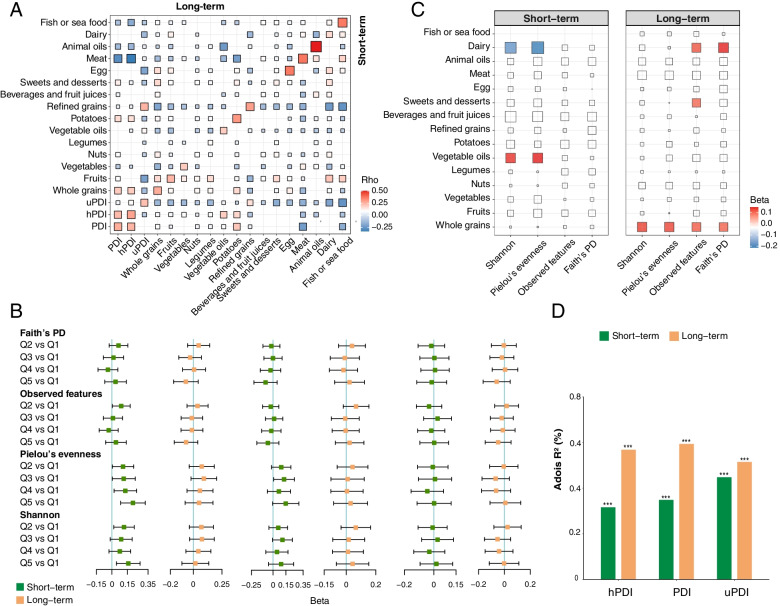
Associations of the long-term and short-term plant-based dietary patterns with overall gut microbiome configuration. **A** Long-term plant-based dietary pattern and its constituent food groups were correlated with short-term estimates. The red-to-blue gradient in the outer blocks represents the magnitude and direction of the Spearman correlation between long-term dietary factors and short-term dietary factors. The correlations were displayed with color within blocks when significant (*p* < 0.05). **B** Associations of long-term and short-term plant-based dietary patterns with bacterial richness and diversity. Beta coefficients were derived from multivariable-adjusted linear mixed models for Q2–Q5 of PDIs using Q1 as the reference group. Covariates included age, sex, BMI, total energy intake, physical activity, education, smoking and alcohol drinking status, habitual tea and coffee consumption, urbanization index, sampling location (random effect), sequencing depth, and sequencing batch (random effect). All four alpha-diversity indices were standardized to *Z*-score values before statistical analysis. **C** Associations of plant-based foods and animal-based foods with bacterial richness and diversity. Beta coefficients were derived from multivariable-adjusted linear mixed models as above for Q5 of each food group using Q1 as the reference group. All four alpha-diversity indices were standardized to *Z*-score values before statistical analysis. Non-significant associations (*p* > 0.05) have been scored 0 and hence colored white. **D** Proportion of variation in taxonomy at ASV level explained by the long-term and short-term plant-based dietary patterns and as quantified by permutational multivariate analysis of variance (based on Bray–Curtis dissimilarity). The *p* values were calculated by adjusting for the same covariates as above. Faith’s PD, Faith’s phylogenetic diversity; hPDI, healthful plant-based diet index; PDI, plant-based diet index; Pielou’s evenness, Pielou’s measure of species evenness; Q, quintile; Shannon, Shannon’s diversity index; uPDI, unhealthful plant-based diet index. *** *p* < 0.001

Long-term PDIs and all 15 constituent food groups were significantly correlated with corresponding short-term estimates (*p* < 0.05, Fig. 2A). The correlation coefficients were 0.26, 0.28, and 0.22 for PDI, hPDI, and uPDI, respectively (Fig. 2A). Among 15 food groups, animal oils (*r* = 0.50), meat (*r* = 0.37), eggs (*r* = 0.36), and fish or seafood (*r* = 0.36) showed strongest correlations, whereas beverages and fruit juices (*r* = 0.08), nuts (*r* = 0.09), and legumes (*r* = 0.09) showed the weakest correlations.

## Plant-based diet and gut microbiome

Higher short-term hPDI was associated with higher Shannon’s diversity index and Pielou’s measure of species evenness (Fig. 2B). However, no statistically significant association was seen between the long-term PDIs and microbial alpha-diversity (Fig. 2B). Of the 15 food groups, we observed a positive association of short-term intake of vegetable oils with Pielou’s measure of species evenness and Shannon’s diversity index (Fig. 2C, Additional file 1 Table S3). In accordance with a previous study [24], a higher long-term intake of whole grains was associated with all four alpha-diversity indices (Fig. 2C, Additional file 1 Table S4). Interestingly, short-term dairy consumption was inversely associated with Pielou’s measure of species evenness and Shannon’s diversity index, whereas long-term dairy intake had a positive association with observed features and Faith’s phylogenetic diversity (Fig. 2C, Additional file 1 Table S3-S4). Similar results were obtained regardless of further adjustment for prevalent hypertension, type 2 diabetes, or related medicine use for the above diet-microbial diversity associations (Additional file 1 Table S5-S6).

We proceeded to identify the links between a plant-based diet and the overall microbial structure (beta-diversity). In multivariable analyses, the association between long-term PDIs and microbial beta-diversity (at ASV level) was stronger than short-term PDIs (Fig. 2D). The strongest association was found between long-term PDI and microbial beta-diversity, explaining 0.6% of the dissimilarities in the gut microbiota structure (*p* < 0.001).

**Fig. 3:**
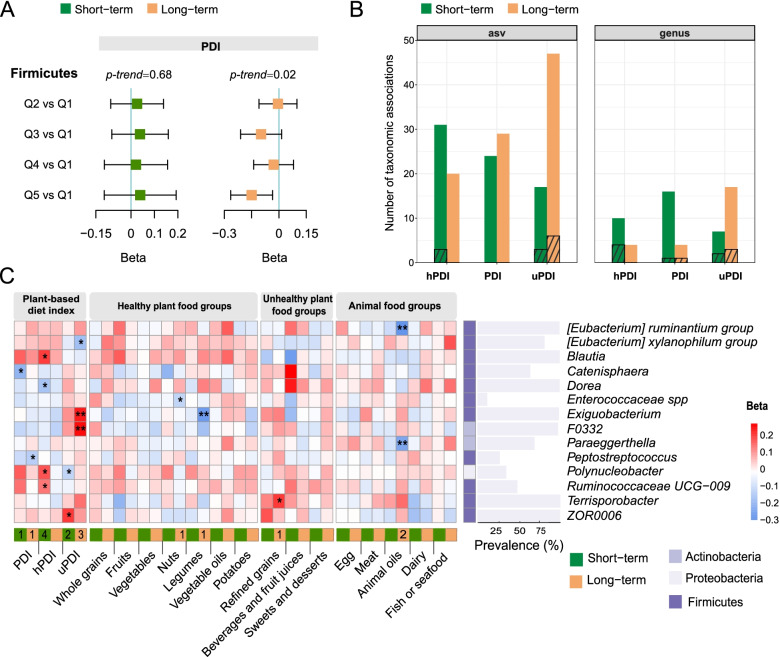
Distinct gut microbial signatures between long-term and short-term plant-based dietary patterns. **A** Association of long-term and short-term PDI with Firmicutes. Beta values were calculated for Q2-Q5 of the PDI using Q1 as the reference group using linear mixed models. All models used sampling location as a random effect and simultaneously adjusted for age, sex, BMI, total energy intake, physical activity, education, smoking and alcohol drinking status, habitual tea and coffee consumption, and urbanization index. The *p*-value for trend was calculated based on per quintile difference in the corresponding plant-based diet index. The relative abundance of microbial taxonomy was transformed using rank-based inverse normal transformation before analysis. **B** The number of nominally significant taxonomic associations observed in genus and ASV level with long-term and short-term plant-based dietary patterns (Q5 vs. Q1 *p*<0.05). The bars with stripes represent those significant after FDR adjustment (*q*<0.25). **C** Associations of long-term and short-term plant-based dietary patterns and its constituent food groups with microbial genera with the asterisks denoting significant associations (FDR *q* < 0.25). Beta coefficients were derived from multivariable-adjusted linear mixed models as above for Q5 of each dietary indicator using Q1 as the reference group. The relative abundance of microbial taxonomy was transformed using rank-based inverse normal transformation before analysis. Microbial genera with no significant associations are not shown. The number of each microbe’s associations with dietary indicators is written within cells when significant. FDR, false discovery rate; hPDI, healthful plant-based diet index; PDI, plant-based diet index; Q, quintile; uPDI, unhealthful plant-based diet index. *FDR *q* < 0.25, ** FDR *q* < 0.05

We then focused on identifying the specific taxa responsible for these diet-microbiota community associations. Higher long-term PDI was associated with lower relative abundance of *Firmicutes* (Q5 vs. Q1 beta =  − 0.15; 95% CI, − 0.26 to -0.03; *p* < 0.05). At the same time, we observed no statistically significant differences in *Firmicutes* across different quintiles of the short-term PDI (Fig. 3A). All three indices based on long-term diet and short-term diet had nominally significant associations with taxonomies at ASV or genus level (Q5 vs. Q1 *p* < 0.05, Fig. 3B). Among 145 genera with a mean relative abundance of ≥ 0.01% and a prevalence of ≥ 10%, a total of 14 genera were significantly associated with at least one dietary indicator (*q* < 0.25). Short-term PDI had one FDR-adjusted significant association, and it was four for short-term hPDI, two for short-term uPDI, one for long-term PDI, and three for long-term uPDI (Q5 vs. Q1 *q* < 0.25, Fig. 3B, Additional file 1 Table S7-S8). We did not find any overlap between the microbial genera associated with long-term PDIs and genera associated with corresponding short-term PDIs (Fig. 3C). We observed an inverse association between short-term PDI and genus *Catenisphaera*, and similarly for long-term PDI and genus *Peptostreptococcus*. Short-term hPDI was positively associated with *Blautia*, *Polynucleobacter*, and *Ruminococcaceae UCG-009*, while inversely associated with *Dorea*. Higher short-term uPDI was associated with a higher relative abundance of *ZOR0006*, but a lower relative abundance of *Polynucleobacter*. Long-term uPDI was positively associated with *Exiguobacterium* and *F0332*, but inversely associated with *[Eubacterium] xylanophilum group*. Under the FDR threshold of 0.15, all the four identified taxonomic associations of the long-term PDIs were still significant, and three taxonomic associations of the short-term estimates were significant (including hPDI-*Polynucleobacter*, uPDI- *ZOR0006* and hPDI- *Blautia*).

In the secondary analyses for specific food groups, we identified five significant diet-microbe associations (Q5 vs. Q1 *q* < 0.25, Fig. 3C, Additional file 1 Table S9-S10). All those significant associations were related to long-term dietary intake. We observed an inverse association between long-term nut intake and *Enterococcaceae *spp., between long-term legume intake and *Exiguobacterium*, between long-term animal oil consumption and *[Eubacterium] ruminantium group* and *Paraeggerthella*. We found a positive association between long-term refined grains intake and *Terrisporobacter*.

In our functional pathway profiling using PICRUST, we did not identify any association of long-term, short-term PDIs or their constituent food groups with microbial functional pathways (Q5 vs. Q1 *q* > 0.25, Additional file 1 Table S11-S14).

## Association of gut microbiota with cardiometabolic risk factors

**Fig. 4:**
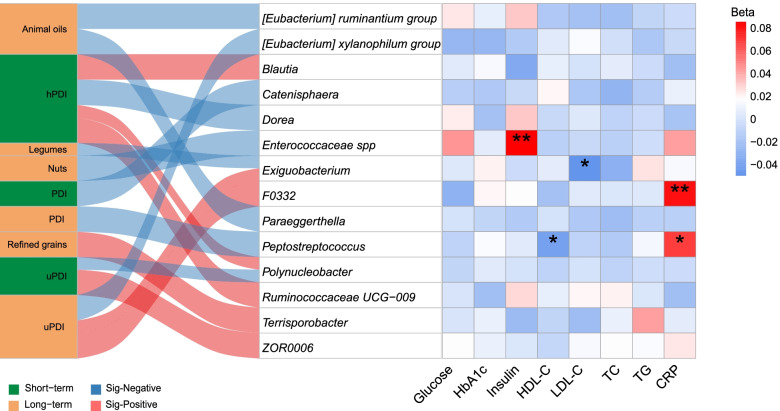
Association of the gut microbial signatures related to plant-based dietary patterns with cardiometabolic biomarkers. The Sankey chart on the left shows the significant microbial signatures of long-term and short-term plant-based dietary pattern and its constituent food groups. The heatmap in the right shows the association between microbial signatures identified and cardiometabolic biomarkers measured after 3 years. Beta coefficients were derived from the linear regression model, adjusted for baseline age, sex, and corresponding cardiometabolic biomarkers measured in 2015 and expressed as the difference in cardiometabolic biomarkers (in standard deviation unit). Correction for multiple testing (FDR) was applied. CRP, high-sensitivity C-reactive protein; FDR, false discovery rate; HDL-C, high-density lipoprotein cholesterol; hPDI, healthful plant-based diet index; LDL-C, low-density lipoprotein cholesterol; PDI, plant-based diet index; Q, quintile; TC, total cholesterol; TG, total triglycerides; uPDI, unhealthful plant-based diet index. * FDR *q* < 0.25, ** FDR *q* < 0.05

After adjusting for potential confounders, we found that higher microbial alpha-diversity (Pielou’s measure of species evenness) were associated with lower TG after 3-year follow-up (*p* < 0.05, Additional file 1 Supplementary Table S15). We also found four gut microbial features (at genus level) of long-term plant diet were significantly associated with future fasting cardiometabolic risk biomarkers, including fasting insulin, HDL-C, LDL-C, TG, and CRP (*q* < 0.25, Fig. 4). Higher long-term PDI was associated with the lower relative abundance of *Peptostreptococcus*, while this microbe was positively correlated with the inflammatory marker (CRP) and inversely associated with HDL-C. *Enterococcaceae *spp., which had an inverse association with long-term nut consumption, and was positively associated with fasting insulin. Higher relative abundance of *F0332* was significantly associated with higher CRP level. However, gut microbial features (at a genus level) related to short-term dietary estimates showed no significant association with future cardiometabolic risk biomarkers.

## Discussion

In the present study, we found that short-term hPDI were associated with microbial alpha-diversity, while long-term PDIs captured more prominent variations in the gut microbial communities (beta-diversity). Long-term and short-term PDIs were differently associated with gut microbial taxonomic composition. Interestingly, only microbes associated with long-term dietary estimates were favorably associated with future cardiometabolic biomarkers. Our results suggested that integrating the short-term and long-term dietary data may help better understand the diet-microbiome associations and suggested the potential utility of long-term plant-based dietary pattern to modulate the gut microbiome and improve cardiometabolic health.

Accumulating literature demonstrates the difference in gut microbiota composition between individuals following vegan or vegetarian diets and those following omnivorous diets [23]. The vegetarian diets including vegan were associated with greater richness of gut microbiota, a greater abundance of *Bacteroidetes*, and a reduced abundance of *Firmicutes* [25]. Some nutrients rich in the plant foods, such as fibers and polyphenols, were strongly associated with gut microbiota [23]. Fibers provide the natural sources for fecal short-chain fatty acids (SCFA), which could alleviate inflammation and show beneficial effects for weight management and type 2 diabetes [26]. Polyphenols are enriched in fruits, vegetables, and seeds and could increase *Bifidobacterium* and *Lactobacillus*, thus increasing the SCFA production and benefiting human health [27]. However, the above evidence is mainly derived from cross-sectional studies using FFQ to capture habitual dietary intake or short-term interventions [9, 24, 28, 29]. The responses of gut microbiota to dietary changes has not been well understood, and many findings are contradictory [28]. Some microbiota are relatively stable over a 32-week dietary intervention [30], while some can rapidly respond to altered diet within 4 days [31].

Therefore, it may be helpful to integrate long-term and short-term dietary assessment in the diet-microbiome association analysis. First, the FFQ was developed to assess long-term habitual intake and is widely used in large epidemiological studies with a relatively low cost [32]. In comparison, repeated 24-h dietary recalls are designed to capture recent dietary intake with relatively higher accuracy [33]. These two methods offer opportunities to integrate long-term and short-term dietary intake for a wide range of diet-microbiome association studies. Second, studies supported that long-term dietary habits exhibited a larger influence on gut community composition [29], while short-term dietary changes showed slight but significant temporary effects [29, 31].

Our results provided different angles to reveal diet-microbiome association with the two types of dietary data (i.e., short-term and long-term dietary assessment). Firstly, we found that only short-term hPDI showed a significantly positive association with microbial alpha-diversity. The inconsistency may result from the dynamic changes of gut microbiota diversity over time which would be substantially affected by many environmental factors [34, 35]. This may attenuate the association of microbial alpha-diversity with long-term plant-based dietary pattern. On the other hand, we provided support that short-term plant-based dietary pattern may be sufficient to alter gut microbial diversity [36]. In terms of specific food groups, we observed contradictory results for the association of short-term and long-term dairy intake with microbial alpha-diversity. Short-term dairy intake was inversely associated with Shannon’s diversity index and Pielou’s measure of species evenness (how evenly the microbes are distributed), whereas long-term dairy intake was positively associated with observed features (how many different microbes in a sample). In a 3-week short-term intervention study, dairy consumption was associated with a decrease in gut microbiota evenness [37]. In addition, habitual long-term dairy intake has been shown to be positively associated with the richness of gut microbiota in another independent cohort [38], consistent with our results. Interestingly, we also observed that long-term whole grain intake was correlated with all four alpha-diversity indices and short-term whole grain intake was not. It suggests that patterns of diet-microbial diversity associations may vary in different food groups, and also fluctuation of microbiota (evenness and richness) to diet within a period of time (long-term and short-term). Taken together, it emphasizes the need to acquire both long-term and short-term dietary data for better understanding of diet-microbiome associations.

Secondly, it was interesting to note that both short-term and long-term PDIs were significantly associated with the overall gut microbiome structure. It justified the use of both habitual dietary intake assessment and recent dietary intake assessment in the diet-microbiome association analysis. Different variations were explained by long-term and short-term estimates. We found that long-term PDIs were more strongly associated with overall gut microbiome structure and the relative abundance of one of the major phyla *Firmicutes*. These results were consistent with several previous studies [23, 39] and indicated the stronger impact of a long-term plant-based diet on the “core” activity of gut microbiota composition. Nevertheless, as there were small variations captured by the long-term PDIs in our study, future long-term intervention studies are needed to validate the above speculation.

We consistently observed positive associations of short-term hPDI with several bacterial genera contributing to carbohydrate fermentation (Table 2). Concordantly, previous studies had demonstrated that the vegetarian diet was associated with the enrichment of pathways related to carbohydrate [28, 40]. Higher proportions of these bacteria have been implicated in cardiovascular diseases through their metabolites such as SCFA [41–43]. *Blautia* was associated with a healthier eating behavior [44], and it could use sucrose and fructose to produce SCFA. Increases in *Blautia* were observed after the administration of high-fiber diet in mice [45]. *Ruminococcaceae UCG-009*, within family *Ruminococcaceae*, could also produce SCFA (acetate and butyrate). *Polynucleobacter* is a propionate producer [43], enriched in healthy athletes [46] and potentially compensates for a disorder in glycogenolysis [47]. Conversely, we saw a negative association of uPDI with *Polynucleobacter* and *[Eubacterium] xylanophilum group*, genera known to produce SCFA and favorably relate to host lipid and glucose metabolism [48–50]. Together, these findings suggest that healthy plant foods may play a role to increase SCFA-producing bacteria and to promote cardiometabolic health and that unhealthy plant foods may have an opposite role. In addition, the consistent associations of long-term and short-term dietary pattern with SCFA-producing bacteria indicate that integrating the short-term and long-term dietary data helps us find more gut microbial signatures of plant-based dietary patterns.

**Table 2:** Overview of diet-microbiome associations in the present study

Findings in the present study		Previous studies	
Genus	Dietary factors^a^	Associations with diet and health	References
*[Eubacterium] ruminantium group*	Long-term animal oil intake ( −)	SCFA-producing bacteria; decrease with an increasing protein/fat in diet; negative association with IL-2 and C-reactive protein	[48]
*[Eubacterium] xylanophilum group*	Long-term uPDI ( −)	SCFA-producing bacteria; inverse association with liver total triglycerides; lower in women who developed gestational diabetes	[48–50]
*Blautia*	Short-term hPDI ( +)	SCFA-producing bacteria; increased after high-fiber diet; linked with healthier eating behavior; negative association with visceral fat, Hb1Ac and inflammation	[41, 44, 45]
*Catenisphaera*	Short-term PDI ( −)	Increase with high-fat diet; decrease with flavonoids intake; enriched in acute coronary syndrome patients; potentially contribute to the inflammation; associated with host lipid metabolism	[51–53]
*Dorea*	Short-term hPDI ( −)	SCFA-producing bacteria; correlated with vegetal protein; higher in patients with irritable bowel syndrome	[54, 55]
*Enterococcaceae *spp.	Long-term nuts intake ( −)	No related information found	
*Exiguobacterium*	Long-term uPDI ( +) Long-term legumes ( −)	Involved in the starch hydrolysis	[56]
*F0332*	Long-term uPDI ( +)	Enriched in children with dental caries; increased abundance in bacterial infection and asthma	[57–59]
*Paraeggerthella*	Long-term animal oil intake ( −)	Involved in ellagic acid metabolism and help produce anti-inflammatory metabolite isolecithine-A	[60, 61]
*Peptostreptococcus*	Long-term PDI ( −)	Involved in tryptophan metabolism; linked with inflammation	
*Polynucleobacter*	Short-term hPDI ( +) Short-term uPDI ( −)	SCFA-producing bacteria; potentially make up for disorders in glycogenolysis; enriched in healthy athletes	[43, 46, 47]
*Ruminococcaceae UCG − 009*	Short-term hPDI ( +)	SCFA-producing bacteria; involved in amino acid metabolism	
*Terrisporobacter*	Long-term refined grains intake ( +)	Opportunistic pathogen; could degrade carbon sources (e.g., glucose, cellobiose, and xylose)	[62, 63]
*ZOR0006*	Short-term uPDI ( +)	Enriched in the fish fed in the paddy field	[64]

^a^( +) positive association; ( −) inverse association

Refined grains are major contributors to daily calorie intake among Chinese population and high refined grain intake has been reported as the leading dietary risk factor for type 2 diabetes [65]. We observed a positive association of uPDI and refined grains with abundance of opportunistic bacterial genera such as *F0332* [57–59] and *Terrisporobacter* [62, 63]. Substituting whole grains for refined grains for 6 weeks has been linked with decreased levels of pro-inflammatory bacteria and SCFA-producing bacteria [66].

Plant-dominated diets tend to be accompanied with lower intake of animal protein and fat, which may substantially affect the intestinal environment and gut microbiome composition [67, 68]. We observed an inverse association of short-term PDI with genera that were increased with high-fat diet (*Catenisphaera*) [51]. We also found an inverse association of long-term animal oil consumption with genera that were decreased with high-fat diet (*[Eubacterium] ruminantium group*) [48]. *Catenisphaera* was associated with higher levels of chronic inflammation and related diseases [51–53], while the opposite direction was reported for *[Eubacterium] ruminantium group* [48]. The above findings together suggest opposite taxonomic associations and health effects for plant-based diet compared with high-fat diet.

Our findings suggest the potential of long-term plant-based dietary pattern for cardiometabolic health promotion through gut microbial metabolism. Long-term PDI was inversely associated with *Peptostreptococcus*, which was positively correlated with an inflammatory marker (CRP). Some species within *Peptostreptococcus* could metabolize tryptophan (one of the amino acids rich in red meat, fish, and eggs) to tryptamine, indolelactic acid, and indolepropionic acid [69]. Those microbial tryptophan metabolites were linked with inflammatory process both in the intestine and in the liver [69, 70]. The genus *F0332* is in the family *Actinomycetaceae* and positively associated with CRP. *Actinomycetaceae* was found to be abundant in the patients with inflammatory clinical phenotypes like chronic bacterial infection and asthma [57, 58]. Such shifts in the gut microbiota population could potentially activate Toll-like receptor signaling pathway and thus increasing intestinal permeability and delivery of pro-inflammatory cytokines into the host circulation [68]. These findings tentatively imply that long-term plant-based dietary pattern may inhibit the pro-inflammatory properties of specific bacteria, thus favoring host cardiometabolic health.

Our study has several limitations. First, the observational nature of the present study cannot unravel causality. Even though we adjust for many confounders in our statistical analyses, we are unable to assess the potential influence of residual confounding. Second, given the sparsity of the gut microbiome data and significance level we use (FDR *q* < 0.25), our study warrants replication in an independent cohort using different statistical methods or a dietary intervention with long-term follow-up. Animal studies may also be needed to uncover the mechanism behind the identified diet-microbiome associations. Third, our analyses are based on genera measured using 16S rRNA sequencing, and thus specific bacterial species and functional genes can not be investigated. Finally, although we use validated questionnaires and standard fecal sampling procedure, the potential measurement error in diet and gut microbiome assessment may still be a concern. Despite these limitations, the present study, to our knowledge, is the first investigation that exclusively elucidates the association of both long-term and short-term plant-based diet with gut microbiota. The use of repeated FFQs and 3-day 24-h dietary recalls allows us to comprehensively assess the long-term and short-term diet. The longitudinal study design also enables us to examine the prospective association between diet-related gut microbial features and cardiometabolic health.

## Conclusions

An integration of short-term and long-term dietary data may help us identify more microbial signatures of plant-based dietary pattern. The present results reveal how the plant-based diet may interact with the gut microbiota features, and provide potential mechanistic insight into the protective role of long-term plant-based dietary pattern for cardiometabolic health.

## Supplementary Information

**Additional file 1: Table S1.** Examples of food items constituting the 15 food groups in the CHNS. **Table S2.** The range of each food group by quintiles. Table S3. Associations of short-term intake of plant-based foods and animal-based foods estimated using 3-day 24-hour dietary recalls in 2015 with bacterial richness and diversity. **Table S4.** Associations of long-term intake of plant-based foods and animal-based foods estimated using repeat food frequency questionnaires in 2011 and 2015 with bacterial richness and diversity. **Table S5.** Associations of plant-based diet indices with bacterial richness and diversity with further adjustment for prevalent hypertension, type 2 diabetes, and related medicine use. **Table S6.** Associations of intake of plant-based foods and animal-based foods with bacterial richness and diversity with further adjustment for prevalent hypertension, type 2 diabetes, and related medicine use. **Table S7.** Associations of short-term plant-based diet indices estimated using 3-day 24-hour dietary recalls in 2015 with bacterial genera. **Table S8.** Associations of long-term plant-based diet indices estimated using repeat food frequency questionnaires in 2011 and 2015 with bacterial genera. **Table S9.** Associations of short-term intake of plant-based foods and animal-based foods estimated using 3-day 24-hour dietary recalls in 2015 with bacterial genera. **Table S10.** Associations of long-term intake of plant-based foods and animal-based foods estimated using repeat food frequency questionnaires in 2011 and 2015 with bacterial genera. **Table S11.** Associations of short-term plant-based diet indices estimated using 3-day 24-hour dietary recalls in 2015 with bacterial pathways. **Table S12.** Associations of long-term plant-based diet indices estimated using repeat food frequency questionnaires in 2011 and 2015 with bacterial pathways. **Table S13.** Associations of short-term intake of plant-based foods and animal-based foods estimated using 3-day 24-hour dietary recalls in 2015 with bacterial pathways. **Table S14.** Associations of long-term intake of plant-based foods and animal-based foods estimated using repeat food frequency questionnaires in 2011 and 2015 with bacterial pathways. **Table S15.** Prospective associations of microbial alpha-diversity with the future cardiometabolic risk factors.

## Data Availability

The datasets used and/or analyzed during the current study are available from the corresponding author on reasonable request.

## Abbreviations

ASV: Amplicon sequence variant
BMI: Body mass index
CHNS: Chinese Health and Nutrition Survey
CRP: High-sensitivity C-reactive protein
FDR: False discovery rate
FFQ: Food frequency questionnaire
HbA1c: Hemoglobin A1c
HDL-C: High-density lipoprotein cholesterol
hPDI: Healthful plant-based diet index
LDL-C: Low-density lipoprotein cholesterol
PDI: Plant-based diet index
PERMANOVA: Permutational multivariate analysis of variance
Q: Quintile
SCFA: Short-chain fatty acids
SD: Standard deviation
TC: Total cholesterol
TG: Total triglycerides
uPDI: Unhealthful plant-based diet index
